# Carotid and popliteal artery intima–media thickness in patients with poor oral hygiene and the association with acute-phase reactants

**DOI:** 10.5830/CVJA-2013-051

**Published:** 2013-10

**Authors:** Ihsan Sami Uyar, Mehmet Besir Akpinar, Veysel Sahin, Feyzi Abacilar, Volkan Yurtman, Faik Fevzi Okur, Elif Filiz Yasa

**Affiliations:** Department of Cardiovascular Surgery, Medical Faculty, Sifa University, Izmir, Turkey; Department of Cardiovascular Surgery, Medical Faculty, Sifa University, Izmir, Turkey; Department of Cardiovascular Surgery, Medical Faculty, Sifa University, Izmir, Turkey; Department of Cardiovascular Surgery, Medical Faculty, Sifa University, Izmir, Turkey; Department of Cardiovascular Surgery, Medical Faculty, Sifa University, Izmir, Turkey; Department of Cardiovascular Surgery, Medical Faculty, Sifa University, Izmir, Turkey; Faculty of Dentistry, Sifa University, Izmir, Turkey

**Keywords:** tooth loss, periodontal disease, infection, atherosclerosis

## Abstract

**Purpose:**

The aim of this study was to evaluate whether poor oral hygiene is associated with carotid and popliteal arterial intima–media thickness, which is one of the predictors of future progression of sub-clinical atherosclerosis, and high-sensitivity C-reactive protein (hsCRP) and fibrinogen levels.

**Methods:**

A specialised dentist checked the patients and selected 550 patients during periodontal examinations, according to their oral hygiene. The patients had no history of atherosclerotic disease. Carotid and popliteal artery B-mode ultrasonographic examinations and hsCRP and fibrinogen levels were analysed at baseline and after a mean of 6.2 months. The patients were scored on the DMFT index for the number of decayed (D), missing (M), and filled (F) teeth (T). We also used the Silness-Loe plaque index (SLI) to evaluate oral hygiene and dental plaque. The patients were divided into two groups using the DMFT and SLI criteria. Group I had a DMFT index score from 0 to 3 and SLI index score of 0 or 1. Group II had a DMFT index score from 4 to 28 and SLI index score of 2 or 3.

**Results:**

A significant association was observed between dental status, oral hygiene, carotid and popliteal artery intima–media thickness and hsCRP level. Patients with increasing DMFT and SLI scores correlated with increasing carotid artery intima–media thickness.

**Conclusions:**

The results clearly showed that chronic poor oral hygiene and tooth loss are related to sub-clinical atherosclerotic changes in the carotid arteries and may be indicative of future progression of atherosclerosis.

## Abstract

Cardiovascular diseases (CVDs) have been the most common cause of death and disability in recent decades. It is not true that conventional risk factors for atherosclerosis account for all atherosclerotic entities, and it has been postulated that novel risk factors such as poor oral hygiene and dental or periodontal disease are potentially associated with atherosclerosis.^1^-^3^

Several studies have reported a close association between CVDs and poor oral hygiene.^4^,^5^ In particular, it has been speculated that chronic inflammation triggered by poor oral hygiene pathophysiologically plays a role in the aetiology of atherosclerosis.^6^,^7^ In this study, we investigated whether poorer oral hygiene and/or periodontal disease indicated sub-clinical atherosclerosis and whether there was any relationship between periodontal disease and poor oral hygiene, and carotid–popliteal arterial intima–media thickness, and hsCRP and fibrinogen levels.

## Methods

In this study, out-patients were evaluated for oral hygiene and dental status at Sifa University Hospital’s School of Dentistry. DMFT index scores, which describe dental status, were obtained by calculating the number of decayed (D), missing (M) and filled (F) teeth (T). We also used the Silness-Loe plaque index (SLI index) to evaluate oral hygiene and dental plaque.^1^-^4^ The mean index was calculated after the evaluation of all teeth and surfaces. A specialised dentist checked patients and decided who could join the study.

Ultimately, 550 out-patients with chronic poor oral hygiene were included in the study. The patients were divided into two groups using the DMFT and SLI index scores as criteria. Group I had a DMFT index score from 0 to 3, no to mild periodontal disease, a SLI index score of 0 or 1, good to mild oral hygiene (*n* = 125; mean DMFT 1.1 ± 1.56; mean SLI 0.56 ± 0.32; 74 males, 51 females, mean age 48.75 ± 9.72 years, range 29–78 years). Group II had a DMFT index score from 4 to 28, severe periodontal disease, a SLI index score from 2 to 3, and poor oral hygiene (*n* = 425; mean DMFT 19.82 ± 6.44; mean SLI 2.76 ± 3.42; 262 males, 163 females, mean age 50.15 ± 9.80 years, range 30–78 years).

The mean follow-up time for all patients was 6.2 months (range 4.1–8.6 months). Patients were assessed after diagnosis, post treatment, and six months later. All patients were treated conservatively for two months with systemic antibiotics and local treatment.

The carotid and popliteal arteries were examined with B-mode ultrasonography, and a carotid or popliteal arterial intima–media thickness of 1 to 2 mm was considered positive, and of up to 2 mm strongly positive for sub-clinical early atherosclerosis. We also measured hsCRP and fibrinogen levels in the blood. An hsCRP level above 3 mg/l and a fibrinogen level above 5 g/l were accepted as significant.

The study also enrolled patients with atherosclerotic carotid and/or popliteal artery disease, as defined by the presence of non-stenotic plaques or carotid stenosis to some degree, who were clinically asymptomatic or symptomatic at the time of screening. For a variety of reasons, 135 patients could not finish the study and were excluded from the study.

Data was collected from the patients between May 2011 and November 2012. Also excluded from the study were patients with diabetes mellitus, high cholesterol or triglyceride levels, hypertension, angina pectoris, a history of myocardial infarction and stroke, a history of rehabilitation, heart surgery, congestive heart failure, peripheral vascular disease, alcohol use, active malignant disease, or any immunological or known chronic inflammatory condition or were currently smoking. The study was approved by the institutional review board of the University of Sifa, and all patients gave signed, written informed consent before enrollment.

## Dental examination

In this study, we used the World Health Organisation-approved dental indices to quantify dental disease: DMFT as a measure of dental status and SLI as a measure of oral hygiene and dental plaque. All dental examinations were performed by specifically trained dentists blinded to the patients’ clinical and ultrasound data. Dental examinations took place one week before the initial ultrasound examination. The oral health parameters of all subjects were recorded at the beginning of the study.

We evaluated all 28 teeth according to the DMFT index, excluding the third molar teeth from the study. DMFT used dental status and the amount of dental caries in an individual to numerically express the caries prevalence. SLI assessed the state of oral hygiene and dental plaque accumulation by measuring both soft and mineralised deposits at four sites per tooth (mesio-buccal, mid-buccal, disto-buccal and mid-lingual). All periodontal findings were taken using the half-mouth method at all four gingival areas of the tooth and marked with a score from 0 to 3. Dental plaque was scored as:

• 0: no plaque
• 1: a film of plaque adhering to the free gingival margin and adjacent area of the tooth, which can be seen in samples from the tooth surfaces
• 2: moderate accumulation of soft deposits within the gingival pocket or the tooth and gingival margin, which can be seen with the naked eye
• 3: abundance of soft matter within the gingival pocket and/or the tooth and gingival margin.

In patients who were completely toothless, SLI was obtained from the prosthesis. SLI of 0 and 1 was defined as absent or mild, and 2 to 3 as serious.

## Carotid B-mode ultrasonography

The carotid and popliteal arterial intima–media thickness and/or stenosis measurements were performed by experienced radiologists using B-mode ultrasonography. The extracranial carotid arteries were examined bilaterally using a 7.5-MHz linear array transducer (Siemens Antares, Germany). The operators were blinded to the patients’ clinical data and dental status.

The patients were in a supine position with their heads turned slightly away from the operator. Measurements were taken in longitudinal and transverse planes on the common carotid artery (CCA) far wall, 1 cm from the bulb, the bifurcation, the internal carotid artery (ICA), external carotid artery (ECA), and popliteal artery. The intima–media thickness was defined as the distance between the leading edges of the lumen–intima echo and media–adventitia echo.

Sub-clinical atherosclerosis was defined as mean carotid and/or popliteal artery intima–media thickness of more than 1 mm, as assessed by B-mode ultrasound. The 1-mm cut-off point was chosen because it has clinical and prognostic significance and has been associated with the subsequent development of coronary artery disease. Carotid plaque was defined as a localised intima–media thickening of more than 1 mm, with at least 100% increase in thickness compared with adjacent wall segments.

If a plaque occurrence was diagnosed during the examination, it defined early atherosclerosis. Plaques were present if they protruded into the lumen or localised roughness with an increased echogenicity or an area of increased thickness of the intima–media layer. Plaque presence was defined as one plaque in any of the carotid arteries. In order to compensate for the stretching effect of arterial distension secondary to increased arterial pressure on the wall thickness, the patients’ systolic arterial pressures were below 140 mmHg.

## Statistical analysis

Continuous data are displayed as means with standard deviation. Categorical data are expressed as proportions. Categorical variables were analysed using the chi-squared test or Fisher’s exact test when appropriate. In all studies, *p* < 0.05 were considered statistically significant.

## Results

The two groups of patients had similar baseline demographics and clinical characteristics. The mean age of the groups was not significantly different (Table 1). Baseline measurements in group II of the mean carotid and popliteal artery intima–media thickness and mean hsCRP levels were significantly higher than that of group I (*p* = 0. 001). However, there was no correlation with dental status, oral hygiene and fibrinogen levels (Table 2). The mean DMFT index scores were 1.1 ± 4.56 in group I and 19.82 ± 6.44 in group II, while the mean SLI index scores were 0.56 ± 2.32 and 2.76 ± 3.42, respectively.

**Table 1:** Demographic And Clinical Data Of The Study Patients

*Parameters*	*Group I, n = 125 (DMFT: 0–3, SLI: 0–1)*	*Group II, n = 425 (DMFT: 4–28, SLI: 2–3)*	p*-value*
Age (mean ± SD)	48.75 ± 9.72	50.15 ± 9.80	0.160
Males, *n* (%)	74 (59.2)	262 (61.64)	0.766
BMI (kg/m^2^) (mean ± SD)	27 ± 4	28 ± 6	0.08
Systolic blood pressure (mmHg) (mean ± SD)	136 ± 21	139 ± 24	0.207
Diastolic blood pressure (mmHg) (mean ± SD)	79 ± 12	78 ± 16	0.51
Family history, *n* (%)	23 (18.4%)	85 (20%)	0.713
Fasting plasma glucose (mg/dl) (mean ± SD)	92 ± 12	94 ± 15	0.172
Total cholesterol (mg/dl) (mean ± SD)	204 ± 28	212 ± 32	0.012
Triglycerides (mg/dl) (mean ± SD)	185 ± 18	187 ± 22	0.35
Uric acid (mg/dl) (mean ± SD)	5.1 ± 2.2	5.2 ± 2.6	0.69
DMFT index (mean ± SD)	1.1 ± 4.56	19.82 ± 6.44	0.001
Mean SLI (mean ± SD)	0.56 ± 2.32	2.76 ± 3.42	0.001
Mean CA IMT (mm) (mean ± SD)	0.63 ± 0.1	1.79 ± 0.14	0.001
HsCRP (mg/l) (mean ± SD)	2.8 ± 3.4	4.7 ± 3.8	0.001
Fibrinogen (g/l) (mean ± SD)	2.9 ± 4.2	4.1 ± 4.4	0.06

Mean ± SD; mean ± standard deviation; BMI, body mass index; DMFT, decayed, missing, filled teeth; SLI, Silness-Loe plaque index; CA IMT, carotid artery intima–media thickness; HsCRP, high-sensitivity C-reactive protein.

**Table 2:** Carotid And Popliteal Arterial Ultrasound Results And Acute-Phase Reactant Levels In Two Groups Of Patients

	*Group I, n = 125 (DMFT: 0–3, SLI: 0–1)*	*Group II, n = 425 (DMFT: 4–28, SLI: 2–3)*	p*-value*
Beginning	*n (%)*	*n (%)*	
Carotid intima–media thickness			
1–2 mm	15 (12)	349 (82.12)	0.0001
> 2 mm	2 (1.6)	76 (17.88)	0.0001
Popliteal artery			
1–2 mm	9 (7.2)	238 (56)	0.0001
> 2 mm	3 (2.4)	183 (43)	0.0001
HsCRP (≤ 3 mg/l)	10 (8)	391 (92)	0.0001
Fibrinogen (≤ 5 g/l)	9 (7.2)	36 (8.3)	0.09
After six months			
Carotid intima–media thickness			
1–2 mm	18 (14.4)	369 (86.82)	0.001
> 2 mm	3 (2.4)	204 (48)	0.0001
Popliteal artery			
1–2 mm	11 (8.8)	259 (60.94)	0.0001
> 2 mm	3 (2.4)	208 (48.94)	0.0001
HsCRP (≤ 3 mg/l)	5 (4)	315 (74.11)	0.0001
Fibrinogen (≤ 5 g/l)	6 (4.8)	26 (6.11)	0.06

DMFT, decayed, missing, filled teeth; SLI, Silness-Loe plaque index; HsCRP, high-sensitivity C-reactive protein.

**Fig. 1.**
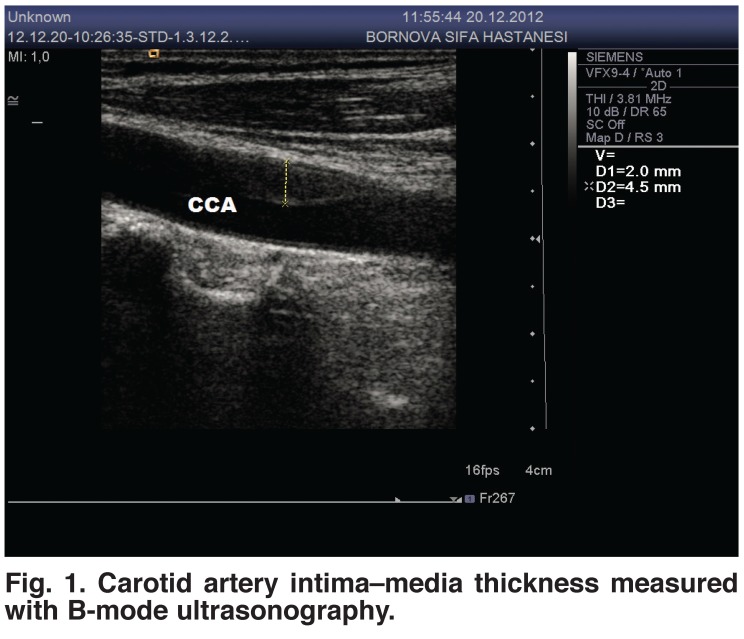
Carotid artery intima–media thickness measured with B-mode ultrasonography.

**Fig. 2.**
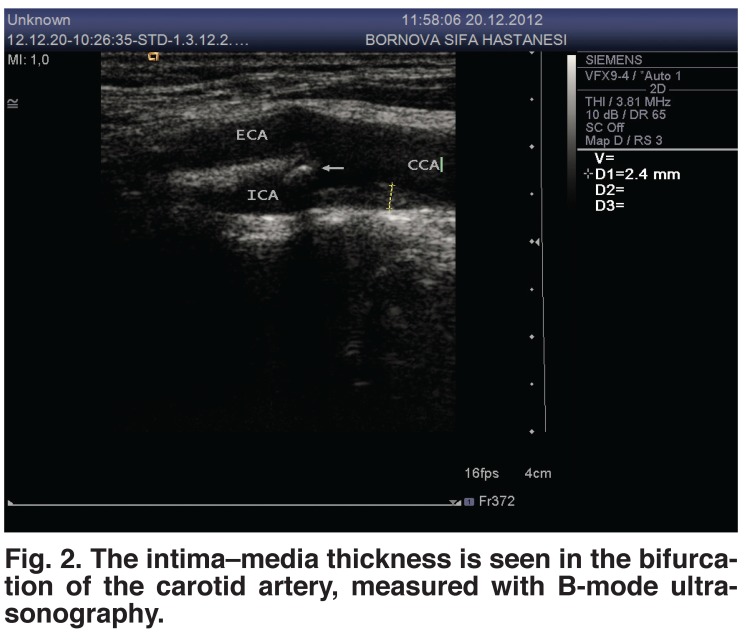
The intima–media thickness is seen in the bifurcation of the carotid artery, measured with B-mode ultrasonography.

Figs 1 and 2 depict carotid artery intima–media thickness measured in B-mode ultrasonography. This examination revealed that patients with an intima–media thickness more than 1 mm numbered 15 (12%) in group I, and 349 (82.12%) in group II (Table 2). This proportion was 92% in patients who were toothless.

Increasing DMFT and SLI scores were correlated with intima–media thickness of the carotid artery. Intima–media thickness was more than 1 mm in 88% of patients with a DMFT index higher than 20, and in 69% of patients with an SLI index of more than 2, but in only 12% of patients with a DMFT index below 3, and in 6.8% of patients with an SLI score of 0 or 1. Significantly more patients with an intima–media thickness greater than 2 mm had a DMFT index more than 20.

The intima–media thickness was greater than 1 mm in 9% of patients with just tooth fillings, significantly lower than the ratio for patients with decayed and missing teeth (*p* = 0.0001). The number of missing teeth was significantly associated with the baseline degree of carotid artery intima–media thickness. Toothless patients also had a significantly higher baseline degree of thickness (*p* = 0.007).

In group II, patients who were completely toothless (11%) and had a dental prosthesis were included as a separate category in the calculations of the SLI. Analysing these patients, we found that toothless patients had a significantly higher risk for disease progression compared to patients in group I (*p* = 0.001).

The hsCRP values were significantly higher in group II than in group I both at baseline and after six months (*p* = 0. 001). The hsCRP levels were higher than 3 mg/l in 8% of patients in group I, and in 92% of patients in group II. The mean levels of fibrinogen were minimally higher in group II than in group I but the difference was not statistically significance (*p* = 0.06). Both DMFT and SLI indices were significantly associated with hsCRP levels at baseline and the six-month follow up. Among patients who received treatment, the intima–media thickness was similar after six months but the hsCRP and fibrinogen levels were significantly lower (*p* = 0.001).

During a mean of 6.2 months (4.1–8.6 months), no deaths were recorded. During the follow-up period, only three patients (2.4%) in group I showed progression of carotid artery intima– media thickness. On the other hand, 47 patients (11%) in group II showed progression of carotid artery intima–media thickness. Patients with progressive atherosclerosis had significantly higher DMFT and SLI indices than patients with stable disease.

Further analysing the sub-categories of DMFT, six-month follow-up measurements revealed that the number of missing teeth was strongly associated with disease progression (*p* = 0.001). However the significance of the number of decayed teeth (*p* = 0.02) and filled teeth (*p* = 0.09) were not significantly associated with disease progression.

## Discussion

Cardiovascular disease is a major cause of morbidity and mortality worldwide. In the last 10 years, a rising number of epidemiological investigations have studied the possible association between inflammatory diseases or chronic infections (i.e. periodontal infections) and cardiovascular diseases.^8^-^10^ According to these studies, atherosclerosis is considered a process closely related to inflammation.

We know that infectious or inflammatory diseases elevate levels of inflammatory markers, and autoimmune processes can contribute to the development of atherosclerosis.^8^ In addition, several studies published during the last two decades indicate that oral diseases, periodontal inflammation, and especially poor oral hygiene may act as risk factors for the development of atherosclerosis via chronic inflammation.^11^-^13^

In particular, chronic microbial infection, including several periodontal pathogens, may play an important role in the development of atherosclerotic disease.^6^,^14^,^15^ It is unclear how periodontal disease causes thickening of the arterial intima–media wall, which is a predictor of sub-clinical atherosclerosis.^16^,^17^ It is uncertain whether an immune response to the pathogens or the pathogen itself triggers progression of atherosclerotic disease.^4^,^18^

We could confirm that missing and decayed teeth were significantly associated with progression of atherosclerosis rather than filled teeth. Treated caries, where the hsCRP level was low, did not seem to play a major role in promoting atherosclerosis. Infectious, poor oral hygiene, a trigger for systemic inflammation, was previously suggested to correlate with carotid intima–media thickness, which is a surrogate marker of atherosclerosis.^2^

Elter *et al.*^19^ proposed a potential mechanism responsible for vascular dysfunction in the presence of periodontal disease. A study by Tonetti *et al.*^20^ concluded that intensive periodontal infection resulted in acute, short-term systemic inflammation and endothelial dysfunction. In addition, periodontal disease has been shown to be a strong predictor of mortality from ischaemic heart disease and diabetic nephropathy in Pima Indians with type 2 diabetes.^21^ Another study postulated that poor oral hygiene may be an insidious cause of endothelial dysfunction and future cardiovascular events.^22^

## Conclusion

The present study has clearly demonstrated a significant relationship between dental diseases, especially tooth loss, and sub-clinical atherosclerosis. We concluded that high DMFT index is associated with increased carotid intima–media wall thickness, and is a marker of early initiation of atherosclerotic lesions. Clinical implications derived from our study are that once a dentist diagnoses advanced dental disease or signs of poor oral hygiene, the patient should be referred to an internist for further screening and, if necessary, the treatment of cardiovascular risk factors.

To demonstrate an association between inflammation, dental indices, poor oral hygiene and disease progression however, longer-term studies are needed. The mean six-month follow-up period was a limitation in this study. Therefore, we will continue to follow the investigation for two years to observe disease progression.

There were other limitations to our study. Pathogen levels or immune responses to the pathogens were not available in our patient population. We could not determine the individual’s propensity to develop inflammatory reaction. Microbial aspects, which have been shown to be more specific than clinical signs of poor oral hygiene, were not evaluated in our study. In addition, the periodontal long-term status was not known. We only investigated clinical measures of dental and periodontal disease.
